# Non-coding RNAs in cancer therapy-induced cardiotoxicity: Mechanisms, biomarkers, and treatments

**DOI:** 10.3389/fcvm.2022.946137

**Published:** 2022-08-23

**Authors:** Wanli Sun, Juping Xu, Li Wang, Yuchen Jiang, Jingrun Cui, Xin Su, Fan Yang, Li Tian, Zeyu Si, Yanwei Xing

**Affiliations:** ^1^Guang'anmen Hospital, China Academy of Chinese Medical Sciences, Beijing, China; ^2^The Second People's Hospital of Jiaozuo, Jiaozuo, China; ^3^Department of Breast Surgery, Xingtai People's Hospital, Xingtai, China; ^4^Beijing University of Chinese Medicine, Beijing, China; ^5^The First Clinical Medical College of Shaanxi University of Chinese Medicine, Taiyuan, China

**Keywords:** non-coding RNAs, cancer therapy, cardiotoxicity, biomarkers, therapeutic strategies

## Abstract

As a result of ongoing breakthroughs in cancer therapy, cancer patients' survival rates have grown considerably. However, cardiotoxicity has emerged as the most dangerous toxic side effect of cancer treatment, negatively impacting cancer patients' prognosis. In recent years, the link between non-coding RNAs (ncRNAs) and cancer therapy-induced cardiotoxicity has received much attention and investigation. NcRNAs are non-protein-coding RNAs that impact gene expression post-transcriptionally. They include microRNAs (miRNAs), long non-coding RNAs (lncRNAs), and circular RNAs (circRNAs). In several cancer treatments, such as chemotherapy, radiotherapy, and targeted therapy-induced cardiotoxicity, ncRNAs play a significant role in the onset and progression of cardiotoxicity. This review focuses on the mechanisms of ncRNAs in cancer therapy-induced cardiotoxicity, including apoptosis, mitochondrial damage, oxidative stress, DNA damage, inflammation, autophagy, aging, calcium homeostasis, vascular homeostasis, and fibrosis. In addition, this review explores potential ncRNAs-based biomarkers and therapeutic strategies, which may help to convert ncRNAs research into clinical practice in the future for early detection and improvement of cancer therapy-induced cardiotoxicity.

## Introduction

In the last decade, significant advancements in cancer treatment have been made, including anthracycline chemotherapy, molecular targeted therapy, and radiotherapy, which have considerably improved the survival rates of cancer patients, lengthened survival time, and enhanced their quality of life. The National Cancer Institute estimates that at least 16.9 million cancer survivors will be alive in the US in 2019 and that the number will be nearly 22.1 million by 2030 (1). Cardiovascular hazards connected with cancer treatment, such as arrhythmia, arterial hypertension, thromboembolic ischemia, myocardial fibrosis, and heart failure, are growing as cancer treatment improves (2, 3). Cardiotoxicity is one of the primary causes of morbidity and death in patients with cancer, posing a severe danger to the treatment's lengthy effectiveness and life quality (4). An observational study comparing the general US population with 3,234,256 US cancer survivors (1973–2012) found that 38.0% of patients died from cancer and 11.3% died from cardiovascular disease (CVD) (5). Furthermore, patients with cancer from diagnosis to surviving cancer (all sites) had a higher risk of dying from CVD compared to the general US population. The current diagnosis and management of cancer therapy-induced cardiotoxicity are hampered by several factors. The link between cancer therapy and cardiotoxicity is complex, especially due to our incomplete understanding of the underlying pathogenesis, resulting in a lack of effective and specific early diagnosis methods and treatment strategies. As a result, it is vital to understand the pathophysiology and underlying molecular processes of cancer therapy-induced cardiotoxicity, so that novel diagnostics and therapeutic targets may be developed.

**Figure 1:**
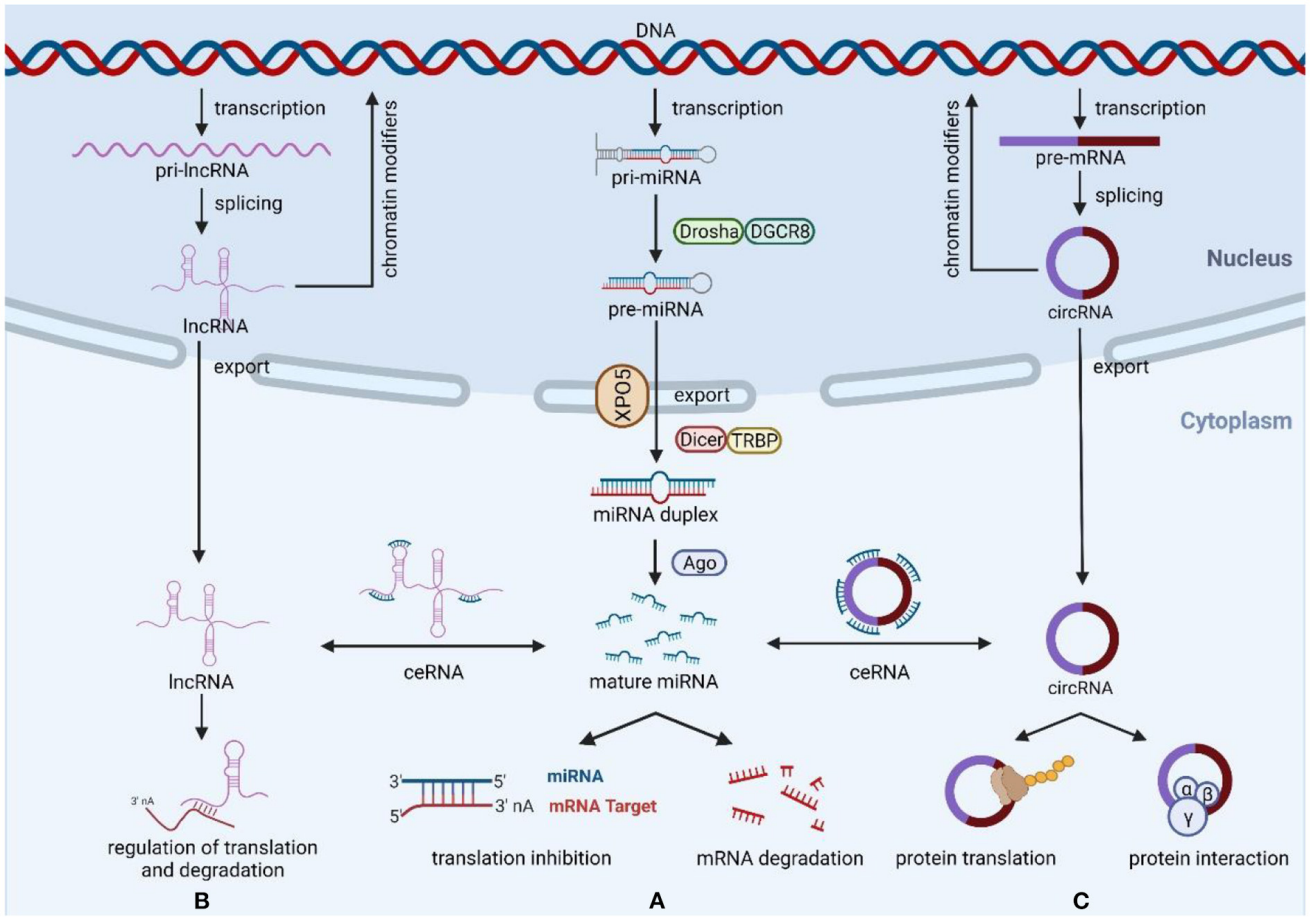
Biogenesis and function of miRNAs, lncRNAs, and circRNAs. **(A)** The ncRNAs genes are transcribed to generate pri-miRNAs, Drosha, and DGCR8 process pri-RNAs to pre-miRNA, then export the pre-miRNAs from nucleus to cytoplasm *via* exportin-5, and generate miRNA duplexes by Dicer and TRBP processing. Mature miRNAs regulate the expression of target mRNAs through degradation or translational repression. **(B)** Most transcribed lncRNAs are polyadenylated at 3′, 5′ capping and splicing. LncRNAs exert their functions mainly through three mechanisms of action, including miRNA spongeization, regulation of translation and degradation, and modifiers of parental gene expression. **(C)** CircRNAs are formed by back-splicing of pre-mRNA, and their main functions include miRNA sponges, protein interactions, protein translation, and regulation of parental genes. Created with BioRender.com.

Non-coding RNAs (ncRNAs) are a class of genetic, epigenetic, and translational regulators, mainly composed of microRNAs (miRNAs), long non-coding RNAs (lncRNAs), and circular RNAs (circRNAs) (6) (Figure 1). MiRNAs are short ncRNAs with a length of 19–24 nucleotides that regulate post-transcriptional gene expression *via* sticking to the 3′ untranslated region (3′-UTR) of target mRNAs and restricting their stability and translation, thus playing a role in a variety of physiological processes including cell development, cell death, proliferation, and signaling (7, 8). Unlike miRNAs, lncRNAs are ncRNAs over 200 nucleotides in length with mRNA-like structures that can control the structure and transcription of the nucleus and influence mRNA stability or serve as competing endogenous RNAs (ceRNAs) interact with miRNAs to regulate mRNA translation (9). CircRNAs are newly discovered ncRNAs whose 5′ and 3′ ends are covalently closed by a back-splicing reaction and have been shown to exert their functions through a variety of mechanisms, including miRNA sponges, protein interactions, protein translation, and parental gene control (10). Because of their important regulatory roles in multiple biological processes of disease development, ncRNAs have great potential as biomarkers and therapeutic targets. Accumulating evidence suggests that ncRNAs have a critical role in the occurrence and development of cardiotoxicity generated by cancer treatment, as well as in pathological conditions such as oxidative damage, mitochondrial damage, apoptosis, dysregulation of calcium homeostasis, and dysregulation of vascular homeostasis (11–13). Therefore, this review summarizes the mechanisms of three ncRNAs (miRNAs, lncRNAs, and circRNAs) in cancer therapy-induced cardiotoxicity and discusses their diagnostic and therapeutic potential in this disease.

## ncRNAs in cancer therapy-induced cardiotoxicity

**Figure 2:**
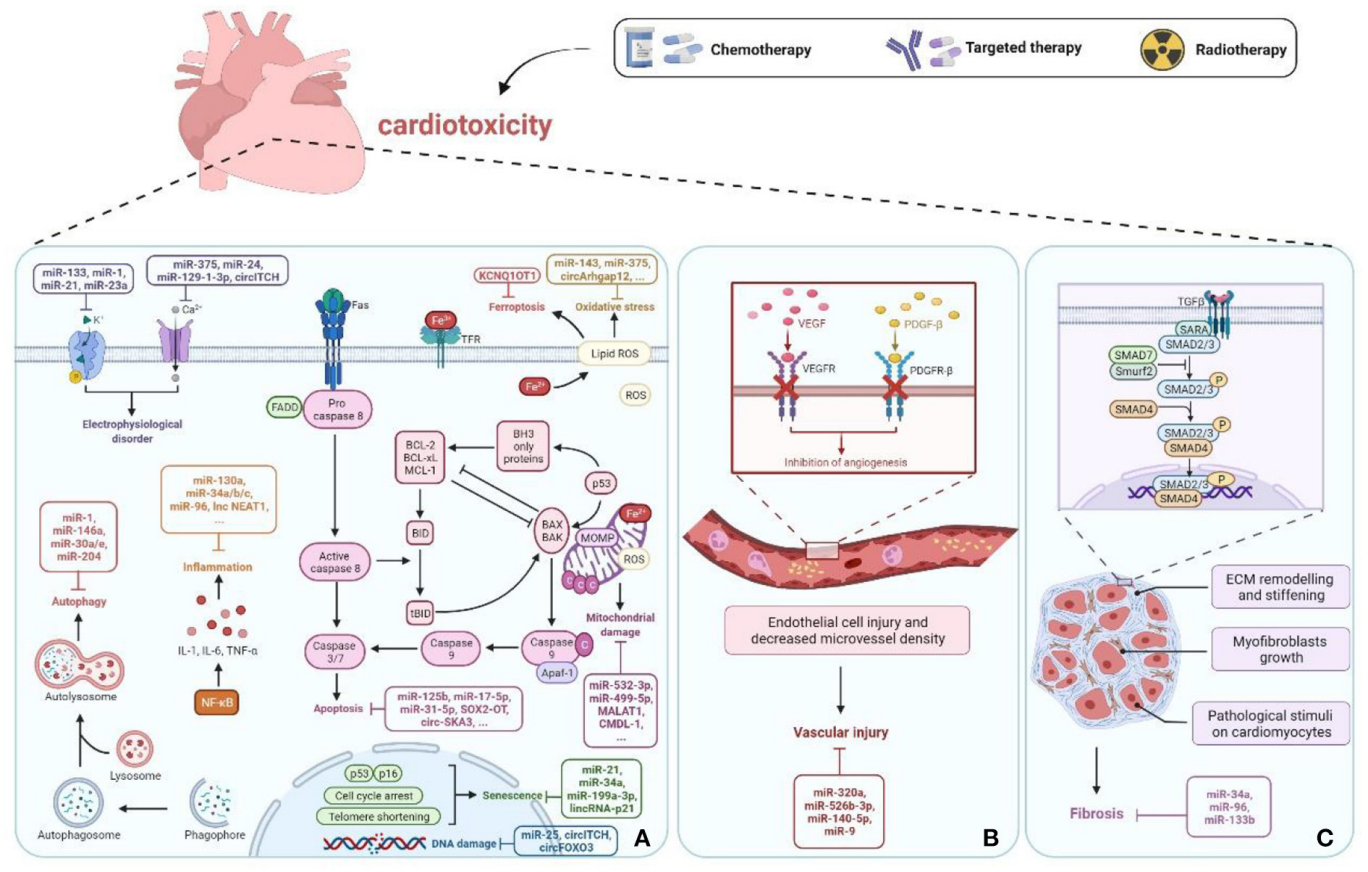
ncRNAs in cancer therapy-induced cardiotoxicity. **(A)** ncRNAs cause cell damage or death through regulation of apoptosis, mitochondrial damage, oxidative stress, DNA damage, inflammation, autophagy, senescence, electrophysiological disorders, and ferroptosis pathways. **(B)** Vascular homeostasis disorder. **(C)** Fibrosis. Created with BioRender.com.

There is increasing evidence that the dysregulation of ncRNAs is associated with cancer therapy-induced cardiotoxicity and plays a crucial role by affecting apoptosis, mitochondrial damage, oxidative stress, inflammation, autophagy, calcium homeostasis, vascular homeostasi, fibrosis, etc. (Figure 2). The molecular mechanisms and functional importance of known ncRNAs implicated in cancer therapy-induced cardiotoxicity are summarized in Table 1.

**Table 1:** ncRNAs in cancer therapy-induced cardiotoxicity.

**Cancer-therapy**	**NcRNAs**	**Expression**	**Target**	**Source**	**Function**	**Reference**
**Anthracyclines**	**ncRNAs**					
**DOX**	**miRNAs**					
	miR-199a-3p	Down	Akt-Sp1/p53	H9c2 cells, mice myocardium	Apoptosis	(14)
		Down	GATA4	hiPSC-CMs, rat myocardium	Cell senescence	(15)
	miR-125b	Up	STARD13 /YAP	Mice myocardium, H9C2 cells	Apoptosis	(16)
	miR-17-5p	Down	PTEN	Mice myocardium	Apoptosis	(17)
	miR-31-5p	Up	QKI/cirPan3	H9c2 cells, mice myocardium	Apoptosis	(18)
	miR-212/132	Down	Fitm2	Mice myocardium, neonatal rat cardiomyocyte and human iPSC-derived cardiomyocytes	Apoptosis, atrophy	(19)
	miR-34a-5p	Up	Sirt1/P66shc	Rat myocardium, H9C2 cells	Apoptosis	(20)
	miR-1	Up	–	Mice myocardium	Apoptosis	(21)
	miR-145	Down	–	Mice myocardium	Apoptosis	(21)
	miR208a, let-7g	–	–	Rat myocardium	–	(22)
	miR-200c	Up	ZEB1	CmPC, mice myocardium	–	(23)
	miR-208a-3p	Up	GATA4	Mice myocardium	Apoptosis	(24)
	miR-146a	Up	ErbB4	Mice myocardium	Apoptosis	(25)
		Down	TAF9b/P53	Mice myocardium, AC16 cells	Autophagy, apoptosis	(26)
	miR-98	Up	Caspase-8/Fas/RIP3	Rat myocardium	Apoptosis, oxidative stress	(27)
	miR-377	Up	–	AC16, mice myocardium	Apoptosis, oxidative stress	(28)
	miR-375	Up	PDK1/AKT	H9c2 cells, mice myocardium	Apoptosis, oxidative stress	(29)
	miR-22	Up	SIRT1	H9c2 cells, mice myocardium	Apoptosis, oxidative stress	(30)
	miR-124	Down	p66Shc	Primary cardiomyocytes, mice myocardium	Apoptosis, oxidative stress	(31)
	miR-143	Up	AKT	H9c2 cells, mice myocardium	Apoptosis, oxidative stress	(32)
	miR-451	Up	AMPK	H9c2 cells, mice myocardium	Apoptosis, oxidative stress	(33)
	miR-200a	Down	Nrf2	H9c2 cells, mice myocardium	Apoptosis, oxidative stress	(34)
	miR-140-5p	Up	Nrf2/Sirt2	H9c2 cells, mice myocardium, rat myocardium,	Oxidative stress	(35, 36)
	miR-15b-5p	Up	Bmpr1a	H9c2 cells	Apoptosis, oxidative stress, mitochondria damage	(37)
	miR-23a	Up	PGC-1α/p-Drp1	Rat myocardium	Mitochondria-dependent apoptosis	(38)
	miR-29b	Down	Bax	Rat myocardium	Mitochondria-dependent apoptosis	(39)
	miR-532-3p	Up	ARC	Rat and mouse cardiomyocytes, Hela cells, human hepatocellular carcinoma cell line HepG-2, human gastric cancer cell line SGC-7901, human colorectal cancer cell line SW-480	Mitochondrial fission, apoptosis	(40)
	miR-499-5p	Down	p21	H9c2 cells, mice myocardium	Mitochondrial fission, apoptosis	(41)
	miR-25	Up	PTEN	Mice myocardium, H9c2 cells, HEK293T cells, DLBCL cell lines (NUDUL−1 and TMD8)	Apoptosis, ROS generation, DNA damage	(42)
	miR-152	Down	Nrf2	Mice myocardium, cultured cardiomyocytes.	Inflammation, oxidative stress, apoptosis	(43)
	miR-200b	Up	Zeb1	Mice myocardium, primary cardiomyocytes	Apoptosis, inflammatory	(44)
	miR-425	Down	RIPK1	Mice myocardium, HL-1 cells	Apoptosis, inflammation and oxidative stress	(45)
	miR-130a	Up	PPARγ	mESCs-derived cardiac cells	Apoptosis, inflammation	(46)
	miR-34b/c	Up	ITCH/NF–κB	Mice myocardium, HL-1 cells	Inflammation	(47)
	miR-30a	Down	Beclin 1	The rat myocardium	Autophagy	(48)
	miR-204	Down	HMGB1	Mice myocardium, H9c2 myocardial cells	Autophagy, inflammation, apoptosis	(49)
	miR-30e	Down	Beclin 1	The rat myocardium	Autophagy	(50)
	miR-34a	Up	PNUTS, Bcl-2/SIRT1	Rat myocardium, H9C2 cells	Apoptosis, senescence, fibrosis, inflammatory	(51–54)
	miR-21	Up	PTEN, BTG2	Neonatal rat cardiomyocytes, H9c2 cells, mice myocardium,	ROS generation, lipid peroxidation, cell senescence, apoptosis	(21, 55–58)
	miR-24	Up	JP-2	The rat myocardium	E–C coupling	(59)
	miR378*	Down	Calumenin	Rat myocardium	ERS–induced cell apoptosis	(60)
	miR-526b-3p	Up	VEGF-A	Mice myocardium, HUVECs	Vascular homeostasis	(61)
	miR-320a	Up	VEGF-A	Mice myocardium, H9c2 and HUVEC	vascular homeostasis	(62)
	miR-133b	Down	PTBP1, TAGLN2	HL-1 cells, mice myocardium	Apoptosis, fibrosis	(63)
	miR-96	Down	Rac1/NF-κB	Rat myocardium, H9c2 cells	Inflammatory, fibrosis, oxidative stress	(64)
THP	miR-22-5p	Down	RAP1/ERK	Rat myocardium, H9C2 cells	Oxidative damage, apoptosis	(65)
	miR-125b-1-3p	Up	JunD	HL-1 cells	Apoptosis, oxidative stress	(66)
	miR-129-1-3p	Down	GRIN2D	H9C2, HL-1 cells	Calcium overload, oxidative stress, apoptotic	(67)
EPI	miR-1	Down	PI3K/AKT/mTOR/NF-κB	Rat cardiac myocytes H9C2, mice cardiomyocytes LH-1, BALB/c mice	Autophagy, apoptosis	(68)
**DOX**	**lncRNAs**					
	PVT1	Up	miR-187-3p/AGO1	H9C2 cells	Apoptosis	(69, 70)
	SOX2-OT	Up	miR-942-5p/DP5	Primary cultured cardiomyocytes, rat myocardium	Apoptosis	(71)
	KCNQ1OT1	Up	FUS	Myce myocardium, HL-1 cells	Apoptosis	(72)
		Up	miR-7-5p/TFRC	AC16, rat myocardium	Ferroptosis	(73)
	CRNDE	Down	PARP-1/HMGB1	Myce myocardium, HL-1 cells	Apoptosis	(74)
	NEAT1	Down	Let-7f-2-3p	Rat myocardium, H9C2 cells	Apoptosis	(75)
	SNHG1	Down	miR-195/Bcl-2	AC16 cells	Apoptosis	(76)
	LINC00339	Up	miR-484	Primary cultured myocardial cell, H9C2 cell	Apoptosis	(77)
	Mhrt	Down	Nrf2	Primary cardiomyocytes, H9C2 cell, rat myocardium	Apoptosis	(78)
	CMDL-1	Down	Drp1	H9C2 cell, rat myocardium	mitochondrial fission, apoptosis	(79)
	MALAT1	Down	miRNA-92a-3p/ATG4a	hiPSC-CMs, human adipose-derived MSCs	mitochondrial metabolism	(80)
	FOXC2-AS1	Down	WISP1	myce myocardium	reduce viability	(81)
	lincRNA-p21	Up	Wnt/β-catenin	HL-1 cells	oxidative stress, cardiac senescence	(82)
**DOX**	**CircRNAs**					
	mmu_circ_0016006, mmu_circ_0015773, mmu_circ_0002106	Up	–	Mice myocardium	Apoptosis	(83)
	circ-SKA3	Up	miR-1303/TLR4	AC16 cells	Apoptosis	(84)
	Ttn, Fhod, Strn3	Down	–	Mice myocardium	Apoptosis, atrophy	(85)
	circArhgap12	Up	miR-135a-5p/ADCY1	Mice myocardium	Apoptosis, oxidative stress	(86)
	circITCH	Down	miR-330-5p	hiPSC-CMs, autopsy specimens from patients with cancer with DOX-treated cardiomyopathy	Oxidative stress, DNA damage, apoptosis, regulation of contractility and calcium handling	(87)
**Radiotherapy**	**ncRNAs**					
	circFOXO3	Up	–	AC16 cells	DNA damage and apoptosis	(88)
	miR-34a	Up	SIRT1	HCMs	Cellular senescence, oxidative stress	(89)
**As**_**2**_ **O**_**3**_	**ncRNAs**					
	lnc NEAT1	Down	miR-124/NF-κB	H9c2 cells	Inflammatory response, apoptosis	(90)
	miR-21	Up	Sp1	hERG-HEK293 cells, rat myocardium	Dysfunction of herg	(91)
	miR-23a	Up	–	hERG-HEK293 cells, rat myocardium	Dysfunction of herg	(91)
	miR-133, miR-1	Up	ERG, Kir2.1	Guinea pig cardiomyocytes	Electrical disorder	(92)
BVZ	miR-140-5p	Up	VEGFA/14-3-3γ	HCMs	Oxidative damage	(93)
PDGFR	miR-9	Up	PDGFR-β	U87 glioblastoma cells, neonatal cardiomyocytes	Angiogenic capacity	(94)

Two miRNAs generated on the two arms of the miRNA precursor, the researchers labeled the miRNAs with low abundance with “*”.

### Anthracyclines

Doxorubicin (DOX), pirarubicin (THP), and epirubicin (EPI) are among the most often used anthracyclines, which are used to treat breast cancer (BC), gastric cancer, acute lymphoblastic leukemia, and lymphoma. However, anthracyclines' clinical use is restricted due to their harmful effects on normal tissues, particularly cardiotoxicity. To date, a large number of studies have confirmed that ncRNAs are abnormally expressed and participate in anthracycline-induced cardiotoxicity by regulating molecular signaling pathways. However, where miRNAs have been extensively studied, lncRNAs and circRNAs are still in their infancy. In-depth knowledge of how these ncRNAs contribute to anthracycline-induced cardiotoxicity will help to identify novel biomarkers or therapeutic targets and provide a more thorough theoretical foundation and evidence base for clinical applications.

#### miRNA

##### Apoptosis

Apoptosis, which is separated into exogenous and endogenous apoptosis, is the best researched programmed cell death route (95). The Fas-involved pathway, which triggers caspase-8, is primarily responsible for exogenous apoptosis. Endogenous apoptosis, on the other hand, is mainly associated with the proapoptotic proteins Bax/Bak of the Bcl-2 protein family, inducing mitochondrial outer membrane permeability and cytochrome c release. Both pathways activate the caspase cascade, which results in cardiomyocyte death. The endogenous apoptotic pathway is currently the most studied in ncRNA- and anthracycline-induced cardiotoxicity. In DOX-treated rat myocardial, for example, miR-34a-5p was upregulated, enhancing Bax expression and mitochondrial depolarization while inhibiting Bcl-2 expression (20). Sirt1, which has miR-34a-5p binding sites in its 3′-UTR, is its downstream mediator. Sirt1 is a NAD-dependent deacetylase that affects mitochondrial function, apoptosis, and inflammatory responses (96, 97). After DOX therapy, Sirt1 expression declined, and its target gene P66shc was negatively regulated, enhancing apoptosis produced by the p66shc-mediated mitochondrial death pathway. As a result, activation of the Sirt1/p66shc pathway is responsible for miR-34a-5p's influence on cardiomyocyte apoptosis. Interestingly, the activation of the NF-κB/p65 signaling pathway was discovered to promote the elevation of miR-34a-5p in DOX-treated myocardial cells, which suggests that the upstream mechanism of miR-34a-5p has to be further researched in the future.

Cardiomyocyte atrophy and apoptosis were increased in DOX-induced human-induced pluripotent stem cell-derived cardiomyocytes (hiPSC-CMs) and primary neonatal rat cardiomyocytes, while upregulation of the miR-212/132 cluster protected against DOX-treated atrophy and apoptosis (19). Upregulation of the miR-212/132 cluster by the adeno-associated virus (AAV) 9 improved cardiac ejection fraction and myocardial wall thickness in DOX mice, alleviating cardiac dysfunction. Overexpression of Fitm2, a downstream target of the pro-hypertrophic miR-212/132 family, partially reversed the anti-apoptotic and anti-atrophic functions of the miR-212/132 cluster, which has been identified as localized in the endoplasmic reticulum and implicated in lipid droplet production. According to a recent study, miR-125b was elevated in DOX-caused cardiomyocytes, and miR-125b suppression diminished apoptosis and ameliorated myocardial damage (16). In DOX-treated cardiomyocytes, miR-125b was increased, and miR-125b inhibition reduced apoptosis and improved cardiac damage. STARD13 was a straight target of miR-125b, which could inhibit the nucleocytoplasmic translocation of YesaSociated protein (YAP) by targeting STARD13. Therefore, miR-125b can play a proapoptotic role in DOX-caused cardiotoxicity by modulating the STARD13/YAP axis, whereas the exact molecular mechanism remains unclear. This study also showed that mesenchymal stem cell-derived small extracellular vesicles (MSC-sEVs) protect the heart against DOX-induced apoptosis, with miR-199a-3p playing a significant role (14). Through the Akt-Sp1/p53 signal pathway, miR-199a-3p in MSC-sEVs upregulated the production of anti-apoptotic proteins survivin and Bcl-2, inhibiting DOX-induced cardiomyocyte apoptosis and improving cardiac contractile function.

##### Mitochondrial damage

Mitochondria are the prominent organelles for DOX-induced cardiomyocyte injury (98). DOX can remain in the mitochondrial inner membrane, creating an irreversible binding with cardiolipin and destroying the normal structure and function of mitochondria. To sustain proper metabolism, the mitochondrial structure undergoes continual fusion and fission, and abnormal mitochondrial fission causes cardiomyocyte death (99, 100). DOX therapy, for example, induced mitochondrial fission and apoptosis in cardiomyocytes *via* upregulating miR-532-3p and adversely controlling the translation of apoptosis repressor with caspase recruitment domain (ARC) in a time-dependent way (40). Overexpression of ARC, on the other hand, prevented DOX-induced mitochondrial fission, reducing cardiomyocyte death. Similarly, miR-499-5p overexpression lowered DOX cardiotoxicity by silencing p21 and inhibiting mitochondrial fission and cell death in myocardial cells (41). Nevertheless, neither of these studies delved into the specific molecular mechanisms by which ARC or p21 control mitochondrial fission.

DOX-related mitochondrial dysfunction is characterized by the loss of mitochondrial membrane permeability and cytochrome c release (101). The cationic drug DOX binds to the negatively charged phospholipids in the inner mitochondrial membrane to form an irreversible DOX cardiolipin complex, which disrupts the electron transport chain (98). Decreased free cardiolipin leads to the detachment of cytochrome c from the mitochondrial membrane, activating caspase-dependent death. In addition, the proapoptotic protein Bax is overexpressed after DOX treatment, and high levels of Bax protein affect mitochondrial membrane permeability by binding to porins on the mitochondrial membrane, promoting mitochondrial release of apoptotic protein cytochrome c, thereby inducing caspase-dependent apoptosis (102, 103). For instance, miR-29b was downregulated in DOX-treated cardiomyocytes and inversely impacted Bax transcription *via* specifically attacking the 3′-UTR of Bax (39). MiR-29b agomir inhibited cytochrome c release, mitochondrial membrane depolarization, and caspase activation while decreasing the proapoptotic protein Bax, lowering mitochondria-dependent apoptotic pathways, and ameliorating DOX-induced cardiac injury. Another study showed that miR-23a and p-Drp1 were significantly increased and PGC-1α was considerably decreased in DOX-treated cardiomyocytes (38). Previous studies have demonstrated that miR-23a directly reduces cardiomyocyte PGC-1α levels by binding to the 3′-UTR of PGC-1α, which can inhibit DOX-induced mitochondrial dysfunction (104, 105). Furthermore, Drp1 is a dynamin-related protein that mediates the mitochondrial outer membrane fission and regulates mitochondrial dynamics (106). MiR-23a inhibitor restored PGC-1α and p-Drp1 levels, which in turn improved mitochondrial membrane potential and inhibited oxidative stress and mitochondria-dependent apoptosis pathways. In contrast, PGC-1α siRNA silencing eliminated the effect of miR-23a inhibitors on PGC-1α and p-Drp1 and the protective effect on cardiomyocytes. Taken together, the knockdown of miR-23a attenuated DOX-induced mitochondrial dysfunction by restoring the PGC-1α/p-Drp1 pathway. Furthermore, Bmpr1a might be a possible target of miR-15b-5p, a crucial regulator in maintaining cardiomyocyte integrity (37). By upregulating miR-15b-5p or suppressing Bmpr1a, DOX-induced oxidative stress, mitochondrial membrane potential damage, and apoptosis might be exacerbated.

##### Oxidative stress

The principal mechanism of anthracycline-induced cardiotoxicity has been considered oxidative stress mediated *via* excess reactive oxygen species (ROS). ROS comprises hydroxyl radical (^·^OH), singlet oxygen (^1^O_2_), superoxide anion (O_2_·-), and hydrogen peroxide (H_2_O_2_), whose excessive production causes lipid peroxidation and structural changes in biomolecules, disrupting the structural integrity of cell membranes and ultimately leading to cardiomyocyte death (107). MiRNA expression is upregulated or suppressed in anthracycline-treated cardiomyocytes, affecting the expression level of their target gene proteins and participating in anthracycline cardiotoxicity owing to oxidative damage, according to several studies. For example, the expression of miR-143 was upregulated after DOX treatment, and miR-143 induced myocardial injury by inhibiting AKT signaling pathway to increase oxidative damage and apoptosis (32). DOX-caused apoptosis and oxidative stress have been demonstrated to be inhibited by activating the AKT signal pathway in previous research (108). Inhibition of miR-143 or activation of AKT reversed DOX-induced myocardial injury. However, the detailed molecular mechanism through which miR-143 and AKT interact is unknown. Conversely, another study confirmed that miR-375 exerted a similar effect by directly targeting 3-phosphoinositide-dependent kinase 1 (PDK1), activating the AKT signaling path (29).

Furthermore, miRNAs have been identified as downstream targets in controlling cardiotoxicity generated by DOX. Nucleolin is a downstream target of miRNA-21, the most numerous RNA-binding protein (RBP) in the nucleolus (56). Following the DOX treatment, the translation of nucleolar protein elevated, as did the expression of miRNA-21, which in turn inhibited ROS generation and lipid peroxidation, thus shielding cardiomyocytes from DOX-induced oxidative damage. This protection could be reversed by knocking down the nucleolar protein. THP-induced cardiotoxicity has been demonstrated to be ameliorated by rutin (RUT), an essential dietary flavonoid (66). After THP treatment, miR-125b-1-3p was upregulated, the JunD expression level decreased, and ROS and apoptosis levels increased in HL-1 cells. JunD, a member of the activator protein-1 transcription factor family, is a key regulator of oxidative stress levels (109). By GO enrichment analysis and TargetScan database screening, a potential miR-125b-1-3p-binding site was found on the 3′-UTR of JunD mRNA. MiR-125b-1-3p may decrease JunD by direct targeting, increasing THP-induced oxidative damage and apoptosis. In contrast, RUT can reverse this effect and thus attenuate THP damage to cardiomyocytes. RUT may also prevent THP-caused cell death and oxidative damage by boosting miR-22-5p expression and blocking the Rap1a-mediated RAP1/ERK signaling pathway, according to a recent study (65).

##### DNA damage

The DNA damage occurs early in DOX-caused cardiac cell death, which might be attributable to direct or indirect ROS generation (110, 111). MiR-25 was shown to be dose-dependently upregulated following DOX treatment, accompanied by an increase in ROS and γ-H2AX (a DNA damage marker) (42). PTEN is a dual lipid/protein phosphatase that has previously been implicated in significantly impacting DNA repair (112). Upregulation of miR-25 causes cardiomyocyte death by interacting with the PTEN 3′-UTR and lowering PTEN levels, resulting in increased ROS generation and DNA damage. Inhibition of miR-25 can restore the expression of PTEN, alleviate DOX-induced cardiotoxicity, and does not affect the anticancer ability of DOX, suggesting that this might be a future therapeutic option for DOX-generated cardiotoxicity.

##### Inflammation

As shown by the increases in proinflammatory cytokines, inflammation response is assumed to be intimately linked to DOX-induced cardiotoxicity (113). Nuclear factor κB (NF-κB), a crucial determinant in the management of inflammation, secretes a variety of proinflammatory cytokines, including interleukin-6 (IL-6), interleukin-1 (IL-1), and tumor necrosis factor alpha (TNF-α) (114). The expression of miR-130a, for example, was dramatically enhanced following DOX therapy and was inversely linked with the expression of PPARγ (46). PPARγ was shown to upregulate the anti-apoptotic protein BCL2, prevent apoptosis, and block NF-κB-mediated cardiomyocyte enlargement, all of which are significant in anti-apoptosis and inflammation (115, 116). Inhibition of miR-130a improved DOX-induced inflammatory response and apoptosis by restoring PPARγ expression, which in turn enhanced BCL2 and lowered NF-κB. MiR-34a,−34b, and−34c are three highly homologous miRNAs that make up the miRNA-34 family. Further research revealed that miR-34b/c increased the secretion of proinflammatory cytokines and aggravated the inflammatory reaction *via* ITCH/NF-κB signaling pathway (47). MiR-34b/c antagomir was targeted to inhibit the expression of miR-34b/c, lower inflammatory cytokine translation, and reverse the cardiotoxicity generated by DOX. AntimiR-34a targeted the inhibition of miR-34a, upregulated Bcl-2 and SIRT1 expression, reduced acetylation levels of p53, SMAD2/3, and NF-κB, as well as reduced inflammatory response, senescence, fibrosis, and apoptosis (51). Furthermore, upregulation of miR-96 alleviated DOX-generated cardiotoxicity *via* blocking the Rac1/NF-κB signal pathway as well as attenuating oxidative stress, inflammatory response, and fibrosis (64).

Inflammation and accumulation of ROS activate caspase-3 and aggravate cell death (101). DOX inhibited the expression of miR-425 and increased the levels of IL-1β, IL-6, TNF-α, and ROS in cardiomyocytes (45). RIPK1 is a Ser/Thr kinase containing a death domain that controls inflammatory signaling and the activation of multiple cell death pathways (117). RIPK1 was found to be a direct target of miR-425 with a miR-425-binding site on its 3′-UTR. MiR-425 overexpression protected cardiomyocytes from DOX-induced inflammation, oxidative stress, and apoptosis by reducing RIPK1 expression. Under normal settings, Nrf2 attaches to the Keap1 protein; however, during stress, it is activated, liberated from Keap1, and binds to antioxidant response elements, controlling various detoxification enzymes and antioxidant genes (118). Overexpression of miR-152 could attach to the 3′-UTR of Keap1 straightly, activating antioxidant and anti-inflammatory actions mediated by Nrf2, as well as reducing cell death (43). The miR-200b production was found to be decreased in cardiomyocytes treated with trophoblast stem cell-derived exosomes (TSC-Exos) in a recent study (44). Myocardial cell inflammatory response and apoptosis were both lowered by TSC-Exos and miR-200b inhibitors. TSC-Exos has anti-inflammatory and anti-apoptotic characteristics and may restore Zeb1 expression by suppressing miR-200b. Nevertheless, the specific method by which TSC-Exos controls miR-200b is uncertain.

##### Autophagy

Autophagy is essential for cardiomyocyte homeostasis, whereas it is disrupted when external stimuli are overwhelming, resulting in cell death (119). Autophagy was dramatically reduced in EPI-treated cardiomyocytes, worsening the apoptotic process (68). Paeonol, a Chinese medicinal extract from Moutan Cortex, can reduce EPI-induced heart damage, boost miR-1 upregulation, and block the PI3K/AKT signal pathway. NF-κB and mTOR are critical downstream targets of PI3K/AKT that significantly impact restoring autophagy levels and reducing myocardial cell death. Another study found that miR-146a, which targeted the TAF9b/P53 pathway, improved autophagy impairment and inhibited apoptosis, ameliorating DOX-induced myocardial injury (26). TAF9b is a co-activator of P53 and mediates autophagy impairment and apoptosis levels by affecting the stability of P53 (120).

Interestingly, recent studies have shown that DOX increases cardiac function impairment by inducing excessive autophagy in cardiomyocytes. The levels of autophagy markers LC3-II/LC3-I and autophagy were raised in DOX-treated rat hearts, while the expressions of both miR-30a and miR-30e were lowered (22, 48). Beclin 1, a downstream target of miR-30, mediates excessive autophagy in cardiomyocytes (121). Enhancing miR-30a/e expression can reduce Beclin 1 translation, inhibit increased cardiomyocytes autophagy, and improve myocardial injury. Another study showed that DOX decreased miR-204 levels *in vivo* and *in vitro*, increased HMGB1 protein expression levels, and promoted the formation of autophagic vesicles in the nucleus (49). HMGB1 is a DNA-binding nuclear protein involved in the regulation of apoptosis/autophagy balance (122). HMGB1 3′-UTR has a miR-204-binding site, and overexpression of miR-204 significantly attenuated DOX-induced autophagy levels and improved myocardial function by directly inhibiting the HMGB1 pathway.

##### Senescence

Cellular senescence has been characterized as a stable cell cycle arrest with the termination of cellular value addition in response to diverse stresses (123). In a DOX-induced cardiomyocyte senescence model, senescence-associated β-galactosidase (SA-β-gal) activity was increased, senescence gene indicators were deregulated, and miR-21 expression was increased (57). The downstream target gene of miR-21, PTEN, has been confirmed. MiR-21 can speed up cardiomyocyte aging by targeting to increase the expression of PTEN, whereas inhibiting miR-21 expression can play an antiaging effect.

The bulk of current research is focused on miR-34a, which has been shown to function on multiple target genes. AntimiR-34a inhibited the expression of miR-34a, which negatively regulated Bcl-2 and SIRT1 expression and delayed cellular senescence (51). SIRT1 is a NAD-dependent sirtuin that mediates cellular senescence by reducing the acetylation level of the senescence-related gene P53 (124). Another study showed that the expression of p16 and p53 was significantly elevated, telomerase activity decreased, telomere length shortened, and cell viability and proliferation decreased after DOX treatment (53). Telomeres are a key marker of cellular senescence, preventing DNA damage at the end of the eukaryotic chromosomes (125). SIRT1 is essential for the elongation of telomeres and the stability of the genome (126). Mesenchymal stem cells (MSCs) can inhibit miR-34a, increase SIRT1 expression, improve telomerase activity and telomere length, restore cell viability and proliferation, and serve as an antiaging agent (53). As a result, MSCs may hold significant therapeutic potential in the therapy of DOX-treated cardiac aging. Furthermore, serum extracellular vesicles (EVs) can delay cardiomyocyte senescence, in which miR-34a plays a vital role (52). Telomere attrition, DNA damage, and apoptosis have been demonstrated to be reduced by PNUTS, a miR-34a target gene (127). Serum EVs inhibit the miR-34a production, which negatively regulates PNUTS expression, thereby playing an antiaging effect.

According to a recent study, miR-199a-3p not only attenuated DOX-induced senescence in cardiomyocytes but also reduced the spread of senescence (15). GATA4 is a critical factor regulating programmed senescence and injury-induced senescence, and miR-199a-3p reduces its translation by binding straightly to its 3′-UTR, which has an antiaging impact. MiR-199a-3p can also inhibit the generation of SASP factors, thus acting as an inhibitor of the spread of senescence. SASP is composed of chemokines, proinflammatory cytokines as well as other factors with deleterious paracrine and systemic effects that contribute to the senescence of otherwise healthy cardiomyocytes (128).

##### Calcium homeostasis

Disruption of calcium homeostasis has been suggested as another critical pathogenic mechanism leading to cardiac injury from anthracyclines (129). Calumenin is a calcium-binding protein located in the endoplasmic reticulum of mammalian tissues and is a crucial participant in calcium ion (Ca^2+^) homeostasis in the endoplasmic reticulum (130). The quantities of calumenin mRNA and protein rose when miR378^*^ was overexpressed. Silencing miR378^*^ inhibited this enhanced expression, suggesting that calumenin might be a miR378^*^ target (60). By inhibiting the expression of calumenin, miR378^*^ may alter endoplasmic reticulum stress and promote the endoplasmic reticulum stress-mediated apoptosis pathway; however, the exact molecular mechanism is still unclear. Another research discovered that suppression of miR-375 alleviated DOX-caused contractile dysfunction *in vivo* and *in vitro* models in which Ca^2+^ has an essential impact on modulating myocardial cell contractility (29). In previous studies, ROS production disrupted cardiomyocyte calcium homeostasis, leading to contractile dysfunction (131). By stimulating the PDK1/AKT signaling pathway, inhibiting miR-375 lowers oxidative damage and enhances heart systolic performance. Furthermore, in cardiac failure caused by DOX, miR-24 expression was considerably enhanced, negatively regulating its downstream factor JP-2 (59). JP-2 is a structural protein that modulates Ca^2+^ signaling by linking the transverse tubule to the sarcoplasmic reticulum, vital for excitation-contraction (EC) coupling (132, 133). The EC-coupling structure was destabilized by the downregulation of JP-2, resulting in decreased Ca^2+^ release and lower myocardial contractility. Inhibition of miR-24 expression and restoration of JP-2 expression can ameliorate DOX-induced heart failure.

In a THP-induced myocardial injury model, the expression of miR-129-1-3p was recently discovered to be downregulated (67). GRIN2D is a subunit of the N-methyl-D-aspartate (NMDA) receptor complex that forms a ligand-gated ion channel with high calcium permeability (134). MiR-129-1-3p negatively modulated GRIN2D expression *via* straightly attaching the GRIN2D 3′-UTR, leading cardiomyocyte Ca^2+^ signaling to become overactive, resulting in calcium overload and cell death. The regulation of calcium homeostasis by overexpression of miR-129-1-3p may offer a novel strategy for treating THP cardiotoxicity.

##### Vascular homeostasis

The maintenance of vascular homeostasis is achieved by the joint action of vascular endothelial cells, fibroblasts, and vascular smooth muscle cells to generate vascular endothelial growth factor (VEGF), nitric oxide, and other vasoactive substances (135). Among them, VEGF-A mediates the development and stabilization of blood vessels, which is essential for maintaining vascular homeostasis (136). Decreased microvessel density and endothelial cell injury lead to the disruption of vascular homeostasis, inducing the development of DOX cardiotoxicity. For example, in DOX-treated mouse hearts, the vascular endothelium was damaged, nitric oxide release, tube formation, and migration of endothelial cells were impaired, the microvessel density markers CD31 and CD34 were decreased, and cardiac microvessels were damaged, accompanied by a significant upregulation of miR-320a (62). Similar to miR-320a treatment, targeting VEGF-A-specific siRNA promotes endothelial dysfunction and perturbs vascular homeostasis. This suggests that VEGF-A is a target of miR-320a and is adversely influenced by miR-320a. MiR-320a inhibition can restore VEGF-A expression, allowing cardiovascular homeostasis to be maintained. Conversely, another study found that miR-526b-3p was associated with DOX-treated dysfunction of vascular homeostasis, but not *via* directly targeting the VEGF-A 3′-UTR to inhibit VEGFA expression (61). This study proposes that miR-526b-3p directly targets STAT3, and the VEGF-A promoter of STAT3 has effective binding sites for BS1 and BS2, to inhibit the transcription of VEGF-A.

##### Fibrosis

Doxorubicin has been linked to cardiac fibrosis in several investigations, and it has been shown to cause fibrous disorganization and accumulation of collagen fibers. DOX-induced ROS production has been reported to trigger the TGF-β route, which stimulates cardiac fibroblasts and promotes fibrosis (137). TGF-β, a pro-fibrotic marker, and its downstream effector, phosphorylated SMAD3, both rose significantly in DOX-induced rat cardiomyocytes (51). Pro-fibrotic and NF-κB-mediated inflammatory factors impact the amount of myocardial fibrosis. AntimiR-34a upregulated Bcl-2 and SIRT1 expression as well as reduced acetylation levels of SMAD2/3 and NF-κB, thus reducing inflammation and fibrosis while also improving cardiac diastolic and systolic function.

Another study showed that miR-96, derived from MSC-Exos, enhanced systolic and diastolic functions as well as attenuated cardiac damage in DOX-treated rat hearts by blocking the Rac1/NF-κB signaling pathway and reducing oxidative stress markers (GSH-Px, SOD, and malondialdehyde), inflammatory cytokines (IL-1β, TNF-α, and IL-6), and collagen fiber accumulation (64). Moreover, overexpression of miR-133b targeted the 3′-UTR of PTBP1 and TAGLN2, negatively regulated their translation levels, and inhibited myocardial cell death and collagen accumulation, thus alleviating DOX-induced myocardial fibrosis (63). PTBP1 belongs to a subfamily of ubiquitously expressed heterogeneous nuclear ribonucleoproteins and is a multifunctional RNA-binding protein involved in many biological processes, including maintenance of cellular structure and motility, immunity, protein metabolism, and the cell cycle (138). TAGLN2 is an actin-binding protein involved in the regulation of cell morphology, motility, and cellular transformation (139). Upregulation of PTBP1 or TAGLN2 may counteract the miR-133b influence on cell death and collagen buildup.

#### lncRNA

##### Apoptosis

The lncRNA Mhrt, which is downregulated in failing cardiac tissue, was first used to investigate the role of lncRNAs in DOX-caused cardiotoxicity (78). Mhrt affects Nrf2 expression levels *via* regulating the formation of the H3 histone and Nrf2 promoter complex. By regulating Nrf2 and enhancing the activity of the proapoptotic protein caspase-3, downregulation of Mhrt induces cardiomyocyte death. By overexpressing the Mhrt-Nrf2 signaling pathway, obstatin was able to reverse these effects. A large body of evidence has demonstrated that lncRNAs might operate as ceRNAs to control miRNA production, therefore participating in DOX-induced apoptosis. For example, a previous study identified LINC00339 as a miR484 target through bioinformatics and luciferase reporter analyses (77). As an endogenous “sponge” of miR484, LINC00339 may be involved in cardiomyocyte apoptosis *via* directly targeting and regulating miR484 production, and its knockdown could reverse the effect on cardiomyocyte apoptosis. Moreover, the production of lnc SNHG1 was diminished in DOX-treated AC16 cells (76). SNHG1 adversely regulated miR-195 production *via* serving as a ceRNA. Bcl-2 was shown to be a miR-195 target gene, known to be a crucial regulator in mediating apoptosis (140). Overexpression of SNHG1 alleviated DOX-induced apoptosis by reducing miR-195 expression, increasing Bcl-2 protein quantities, and inhibiting caspase-3 cleavage.

Similarly, the lncRNA NEAT1 was linked to apoptosis and its expression was lowered considerably following DOX therapy (75). Let-7f-2-3p expression was elevated by the lncRNA NEAT1, which was discovered to be an endogenous sponge RNA of let-7f-2-3p. XPO1 is critical for controlling nuclear protein export, which distributes various anti-apoptotic proteins such as HAX-1 from the nucleus to the cytoplasm to regulate apoptosis (141, 142). Let-7f-2-3p inhibits XPO1 production through targeting its 3′-UTR, inhibiting XPO1-mediated nuclear export of HAX-1, and increasing myocardial enzyme release and cell death. DOX-induced myocardial damage was attenuated without compromising its anticancer activity when NEAT1 was overexpressed or let-7f-2-3p was knocked down. Furthermore, lnc SOX2-OT and DP5 were significantly upregulated after DOX treatment, whereas miR-942-5p was considerably downregulated (71). SOX2-OT acted as a miR-942-5p sponge, affecting its production. MiR-942-5p negatively regulated DP5 expression, an apoptosis-related tumor suppressor gene. Therefore, SOX2-OT suppression diminishes myocardial cell death and prevents cardiac function damage *via* modulating the miR-942-5p/DP5 axis.

Chinese herbal extracts have been demonstrated to defend against cardiotoxicity caused by DOX *via* modulating specific lncRNAs in a recent study (69). Salvianolic acid A (SalA), the effective constituent of Chinese medicine Salvia miltiorrhiza, has an apoptotic inhibitory effect by blocking NFKB1 transcriptional activation and downregulating lncRNA PVT1 production. DOX was reported to enhance PVT1 expression, decrease cell viability, and aggravate apoptosis rate (70). Through adsorbing miR-187-3p *via* sponge action, PVT1 lowered its level. AGO1 is a well-known cell death regulator that controls cell death through numerous pathways (143, 144). AGO1 is a downstream target of miR-187-3p whose expression levels are inversely linked. As a result, the highly expressed PVT1 induced cardiomyocyte death by enhancing AGO1 expression through sponge adsorption of miR-187-3p.

##### Mitochondrial damage

Hypoxia treatment of human adipose-derived MSCs resulted in the accumulation of the lncRNA MALAT1 in secreted exosomes (80). Silencing MALAT1 in MSCs or overexpressing miR-92a-3p in myocardial cells increased Fabp3, Fabp4, and Mtfp1 expressions while decreased Cox4i2, Hspa1a, and Atp1b2 expressions, resulting in mitochondrial metabolic disorders. MALAT1 was discovered to be an exosomal transfer RNA that inhibits the production of miR-92a-3p, which targets and binds to the 3′-UTR of ATG4a. MALAT1 is a ceRNA that binds to miR-92a-3p and activates ATG4a, thereby enhancing mitochondrial metabolism, restoring cardiomyocyte viability, promoting rejuvenation, and inhibiting DOX-induced cardiac aging, according to this study.

A recent study showed that among the lncRNAs with differing expressions, CMDL-1 was the most dramatically attenuated lncRNA in myocardial cells following DOX injection (79). Drp1 is a cytoplasmic protein whose function is decreased and mitochondrial fission is prevented by phosphorylation of its serine residue at position 637 (145). Drp1-mediated mitochondrial fission and apoptosis may be inhibited by the upregulation of CMDL-1, which regulates Drp1 phosphorylation. Drp1 knockdown partially suppressed CMDL-1's anti-mitochondrial fission and anti-apoptotic actions, but not completely. This suggests that CMDL-1 might control Drp1's function rather than transcription; however, the exact molecular mechanism remains further studied.

##### Oxidative stress and senescence

In the DOX cell model, lincRNA-p21 is thought to regulate oxidative stress and may be involved in cellular senescence by inducing ROS production and oxidative damage (82, 146). In HL-1 cells treated with DOX, enhanced lincRNA-p21 expression was accompanied by decreased mitochondrial transmembrane potential and SOD activity, as well as increased ROS production and MDA activity. Moreover, the age-related genes p16 and p53 were upregulated, and telomere length and telomerase activity were decreased. Antioxidant treatment inhibited oxidative stress, reduced ROS production, improved cell viability, and delayed cellular senescence considerably. Similarly, knocking down lincRNA-p21 also helped to reduce oxidative stress and alleviate DOX-induced cardiac aging. The Wnt/β-catenin signal pathway is known to have a role in heart regeneration and aging, and knocking down lincRNA-p21 exerts cardioprotection by activating the Wnt/β-catenin signal pathway (147).

##### Ferroptosis

Ferroptosis has been identified as a unique cell death process that is iron-dependent rather than apoptosis and is defined by a substantial intracellular accumulation of free iron and lipid ROS (148). In the DOX cell model, the lncRNA KCNQ1OT1 is a crucial lncRNA involved in the mechanism of ferroptosis (73). In DOX-treated cardiomyocytes, free iron and lipid ROS were dramatically increased, and METTL14 was upregulated and catalyzed the m6A modification of KCNQ1OT1. MiR-7-5p has been discovered as a direct site of KCNQ1OT1, which binds to the 3′-UTR of the transferrin receptor (TFRC) and controls its expression. TFRC is required for cellular uptake of the transferrin-iron complex, and its overexpression promotes iron uptake and lipid ROS production, ultimately leading to cardiomyocyte ferroptosis (149). Interestingly, miR-7-5p was also involved in the targeted repression of METTL14 expression, implying the existence of an additional feedforward mechanism in the ferroptosis mechanism. In conclusion, the METTL14/KCNQ1OT1/miR-7-5p/TFRC axis plays an essential role in DOX-induced ferroptosis.

#### CircRNA

##### Apoptosis

Qki5 is known to be an RBP that governs various circRNAs (150). According to previous studies, Qki5 regulates a group of circRNAs produced by the genes Fhod3, Ttn, and Strn3, which mediate cardiomyocyte apoptosis (85). MiR-31-5p was discovered to adversely affect the development of circPan3 by directly targeting QKI in recent research (18). By silencing QKI and upregulating circPan3, inhibition of miR-31-5p alleviated apoptosis. However, the downstream molecular processes of circPan3 were not investigated further in this study.

CircRNAs can behave as miRNA sponges, generating anti-apoptotic effects, according to another research. For example, the expression of circ-SKA3 and TLR4 was enhanced following DOX treatment (84). In contrast, the expression of miR-1303 was lowered, which was followed by reduced cell viability and increased proapoptotic proteins. The expression of TLR4 was mediated by Circ-SKA3, which behaved like a sponge for miR-1303. TLR4 is known to belong to the toll-like receptor (TLR) family, which regulates inflammatory and immune responses (151). Circ-SKA3 knockdown attenuated DOX-induced cardiotoxicity *via* the miR-1303/TLR4 axis. Interestingly, extracellular circ-SKA3 was revealed to be bundled into exosomes in this investigation. Exosomal circ-SKA3 may be taken up and internalized by target cells AC16, potentially affecting the RNA expression and function of AC16 cells. Moreover, mmu_circ_0002106, mmu_circ_0016006, and mmu_circ_00115773 were upregulated after DOX treatment, suggesting that these three circRNAs may contribute to myocardial injury by promoting apoptosis, albeit the exact process has to be investigated further (83).

##### Oxidative stress

CircITCH, an E3-ubiquitin protein ligase that modulates tumor suppressor stability, is engaged in oxidative stress generated by DOX (152). In hiPSC-CMs treated by DOX and cancer sufferers' postmortem tissues with DOX heart disease, circITCH expression has been reported to be downregulated (87). CircITCH functions as a natural sponge for miR-330-5p, targeting and regulating it, which in turn controls the interaction between miR-330-5p and the 3′-UTR of SIRT6, BIRC5, and ATP2A2 mRNAs. As previously documented, SIRT6 regulates oxidative stress and DNA damage, BIRC5-encoded survivin regulates apoptosis, and ATP2A2-encoded SERCA2a regulates cardiomyocyte contractile performance and calcium regulation (153–156). CircITCH overexpression ameliorated DOX-induced cardiac dysfunction through modulating the miR-330-5p-SIRT6/BIRC5/ATP2A2 axis, whereas CircITCH knockdown abolished these effects. CircITCH is a broad-spectrum tumor suppressor that also alleviates DOX-induced cardiotoxicity, suggesting that overexpression of CircITCH might be a valuable method to cure DOX-generated cardiotoxicity.

Another study showed that circArhgap12 expression was elevated and ROS generation increased after DOX treatment, which led to cardiomyocyte death (86). CircArhgap12 served as a sponge for miR-135a-5p and inhibiting it alleviated oxidative stress and cell death by controlling miR-135a-5p production. Furthermore, ADCY1 mRNA may contain possible target locations for miR-135a-5p, and the downstream molecular mechanism must be investigated further.

### Radiotherapy

Radiation therapy provides benefits in terms of lowering cancer recurrence and mortality, but at the same time leads to an increased risk of cardiovascular dysfunction (157). It has been reported that miR-34a is significantly upregulated in cardiomyocytes after exposure to radiation, with increased expression of aging-related genes Cdkn1a and Cdkn2c and decreased length and activity of telomere (89). Macrophage migration inhibitory factor (MIF) is a multifunctional cytokine found throughout the body that plays a significant role in cardio-metabolism (158). Exogenous MIF significantly inhibited the radiation-induced expression of miR-34a, which in turn restored SIRT1-mediated antiaging properties. It is well known that SIRT1 is a miR-34a target that has been associated with cellular senescence (124). Oxidative stress hastens the aging of cardiomyocytes (159). Further research revealed that MIF inhibited cellular aging by preventing radiation-induced cardiomyocyte ROS and MDA production *via* regulating oxidative stress. MIF's antioxidant action was abolished when miR-34a was overexpressed or SIRT1 was knocked down. As a result, by regulating the miR-34a/SIRT1 signaling pathway and avoiding oxidative stress-mediated cellular senescence, MIF might be crucial in treating the radiation-induced cardiac injury.

Additionally, circFOXO3 was remarkably upregulated after radiation to promote DNA repair and limit the apoptosis rate to protect cardiomyocytes (88). The effects were abolished when circFOXO3 was knocked out, but the specific chemical mechanism remains unclear.

### Arsenic trioxide

Although arsenic trioxide (As_2_O_3_) is good therapy for acute promyelocytic leukemia and other malignant tumors, its broad clinical applicability is limited due to cardiotoxicity adverse effects (160). In As_2_O_3_-treated guinea pigs, there was a dose-dependent lengthening of the QRS complex and QT interval, which resulted in increased mortality (92). Further studies discovered that As_2_O_3_ upregulated miR-133 and miR-1 production while decreasing ether-a-go-go-related gene (ERG) and Kir2.1 protein levels. Kir2.1 is a potassium channel subunit that is essential to sustain the voltage of the resting membrane and create the final repolarization. It predominantly mediates the inward rectifying potassium current IK1 (161). Long QT syndrome is known to be caused by reduced IKr current density and human ERG (hERG) channel production in As_2_O_3_-treated patients (162). Another research demonstrated that miR-21 and miR-23a inhibited hERG expression and thereby caused As_2_O_3_-induced electrophysiological disturbances (91). As_2_O_3_ increased NF-κB phosphorylation, which upregulated miR-21 and inhibited the expression of Sp1, a transcription factor required for driving transcription from the ERG promoter (163). Additionally, lncRNA NEAT1 plays a crucial role in As_2_O_3_-induced cardiotoxicity (90). By blocking the miR-124/NF-κB signal path and reducing inflammatory response and apoptosis, NEAT1 overexpression protects cardiac cells against As_2_O_3_ injury.

### Targeted molecular drugs

Bevacizumab (BVZ), a monoclonal antibody targeting VEGF ligands, achieves antitumor effects by suppressing tumor angiogenesis and lowering neovascularization. However, cardiotoxic adverse effects may occur after long-term treatment (164, 165). BVZ causes cardiomyocyte mortality that is dose- and duration-dependent, leading to cardiac damage, according to a recent study (93). Previously, miR-140-5p was thought to have a role in DOX-caused oxidative stress (35, 36). After BVZ treatment, miR-140-5p was upregulated and reduced VEGFA expression by targeting its 3′-UTR. The expression of 14-3-3γ, which is linked to oxidative stress and apoptosis in cardiomyocytes, was considerably reduced when VEGFA expression was silenced (166). MiR-140-5p inhibition, which targets the VEGFA/14-3-3γ signaling pathway, lowered MDA and ROS levels and mitigated BVZ-induced oxidative damage. Cardiotoxicity has also been connected to antitumor medicines that target the platelet-derived growth factor receptor (PDGFR). MiR-9 has been reported to regulate PDGFR-β-mediated compensatory angiogenesis by targeting the 3′-UTR of PDFGR-β and adversely regulating its expression (94).

## ncRNAs as biomarkers in cancer therapy-induced cardiotoxicity

Non-coding RNAs have been identified as prospective novel biomarkers for early identification of cardiotoxicity generated by cancer treatment, in addition to the functions and mechanisms of ncRNAs outlined above. Table 2 shows a list of potential ncRNA biomarkers.

**Table 2:** ncRNAs as biomarkers in cancer therapy-induced cardiotoxicity.

**Cancer-therapy**	**ncRNAs**	**Expression**	**Source**	**Reference**
Anthracyclines	miR-423-5p, miR-34a-5pa, miR-126-3p, miR-199a-3p	Up	BC patient serum	(167)
	miR-100-5p, miR-103a-3p, miR-142-3p, miR-143-3p, miR-145-5p, miR-146a-5p, miR-150-5p, miR-181a-5p, miR-199a-5p, miR-29c-3p, miR-320a, miR-342-3p, miR-107, miR-499a-5p, miR-210-3p, miR-92a-3p, miR-486-5p	–	Serum of pediatric patients with malignancy	(168)
	miR-29b, miR-499	Up	Serum of patients with cancer under the age of 18	(169)
DOX	miR-107, miR-146a	Down	Sarcoma dog serum	(170)
	miR-181d, miR-502	Up	Sarcoma dog serum	(170)
	miR-1-3p, miR-122-5p, miR-127-3p, miR-133a-3p, miR-215-5p, miR-455-3-p, miR-499a-5p	Down	Mouse plasma	(171)
	miR-34a-5p	Up	Mouse plasma	(171)
	miR-1	Up	BC patient serum	(172)
	miR-187-3p, miR-182-5p, miR-486-5p, miR-34a-3p, miR-486-3p, miR-212-3p, miR-4423-3p, miR-139-5p, miR-34c-3p, miR-34c-5p	Up	hiPSC-CMs	(173)
	miR-3911, miR-675-5p, miR-4298, miR-1303	Down	hiPSC-CMs	(173)
	miR-34a	Up	Mouse heart	(174)
	miR-208b, miR-216b, miR-215, miR-34c, miR-367	Up	Rat heart	(175)
EC-D	miR-17-5p, miR-20a	Down	BC patient serum	(176)
Radiotherapy	lncRNA Abhd11os, Pvt1, Trp53cor1, Dino, miR-149-3p, miR-6538, miR-8101, miR-7118-5p, miR-211-3p, miR-3960	Up	Mouse heart	(177)
	miR-100-5p, miR-106b-5p, miR-145-5p, miR-146a-5p, miR-192-5p, miR-195-5p, miR-223-3p, miR-25-3p, miR-34a-5p, miR-574-3p, miR-885-5p, Iet-7c, miR-200b-3p, miR-134	–	NSCLC patient plasma	(178)
	miRNA-146a,−155,−221,−222	Up	BC patient blood	(179)
BVZ	miR-30c	Up	NSCLC patient plasma	(180)
	miR1254, miRNA579	Up	Colorectal cancer plasma	(181)

### Anthracyclines

Earlier studies focused on animal models' screening for differential miRNA expression in DOX-induced cardiac tissue. For instance, one study showed that 24 miRNAs were differently expressed in DOX-induced mouse hearts, with miR-34a being the only miRNA significantly upregulated across all DOX doses and miR-34a expression level increasing with DOX dosages overall (174). Furthermore, the lowest cumulative DOX dose that caused miR-34a elevation was 6 mg/kg, which occurred well before cardiac troponin T (cTnT) changes and histopathological damage. In hiPSC-CMs treated with DOX, another study corroborated the early elevation of miR-34a-3p (173). According to these findings, a rise in miR-34a might be a precursor to DOX-induced cardiac injury. Early miRNA changes in the heart may be useful in developing biomarkers to identify patients with DOX-induced cardiotoxicity. However, the invasive nature of tissue biopsy limits its use in clinical research.

Due to the stable expression of miRNAs in nearly all body fluids (blood, serum, saliva, etc.) and the development of detection techniques, circulating miRNAs (c-miRNAs) have recently been viewed as promising early biomarkers for the detection of cardiotoxicity induced by cancer therapy (182). Although miR-208a expression is known to be reduced in DOX-damaged mouse hearts, plasma miR-208a could not be detected in research on DOX-caused BC individuals, even when troponin levels were at their peak after 12 weeks, implying that DOX-damaged hearts do not release miR-208a into the bloodstream (172, 175, 183). MiR-34a-5p expression was upregulated in DOX-treated mice's plasma, whereas seven miRNAs, including miR-133a-3p and miR-1-3p, were suppressed. In this investigation, however, no plasma levels of cardiac troponin I (cTnI) or brain natriuretic peptide were detected (171). Another research in DOX-treated rats found increased expression of plasma miR-133b, miR-1, and miR-133a 24 h following the administration (184). However, there were no noticeable changes in circulating cTnI and cTnT levels or histopathology. It is possible that DOX has not yet caused cardiac injury in rats, and that the acute toxic effects of DOX occur in skeletal muscle before cardiac injury. The circulating levels of miR-1 and−133b were shown to be higher in BC individuals treated with DOX in a study (172). Taken together, it may suggest that varied expression of c-miRNAs is connected to the differences in experimental animal models, species, and levels of DOX treatment and that changes shown in animal models are not always transferable to the human clinical setting.

In recent years, the bulk of studies on c-miRNAs profiles has focused on BC individuals undergoing anthracycline therapy. For instance, four congestive heart failure-related c-miRNAs (miR-423-5p, miR-34a-5p, miR-126-3p, and miR-199a-3p) were identified in patients with BC treated with anthracyclines, whereas another systematic analysis investigated five c-miRNAs (miR-126, miR-210, miR-20a, Let-7f, and miR-1) (167, 176). In a study of epirubicin/cyclophosphamide followed by docetaxel (EC-D)-induced cardiotoxicity in BC individuals, miR-17-5p and miR-20a expression levels were lowered (176). The two miRNAs have a good forecast value for predicting a lower risk of cardiotoxicity, according to receiver operating characteristic (ROC) curves. These findings potentially point to miRNAs as possible biomarkers for predicting anthracycline-induced cardiotoxicity in patients with BC.

Furthermore, heart-related plasma miR-29b and miR-499 levels were considerably elevated 6–24 h following the anthracycline dosing in children and young patients with a drug dose–response relationship, which may facilitate early diagnosis of myocardial injury (169). Consistent with the results, another study showed substantial differences in the levels of 17 miRNAs in children receiving anthracycline chemotherapy in comparison with normal children (168). Among them, miR-499a-5p and miR-29c-3p were found to be considerably elevated following the beginning and finish of anthracycline treatment, respectively.

In a dog sarcoma model receiving only DOX treatment, four chosen EV-miRNAs appeared variably expressed (miR-181d, miR-502, miR-107, and miR-146a) (170). Further studies revealed that miR-181d was upregulated in individuals with a low left ventricular ejection fraction, and miR-502 was upregulated after only two doses of DOX, far ahead of changes in cTnI and echocardiographic parameters. These results may imply that EV-miRNAs can be employed as the biomarkers for early cardiotoxicity identification, although larger sample sizes are needed for future validation.

### Radiotherapy

In patients with BC treated with radiotherapy, the production of miRNAs-221,−222,−146a, and−155 in the blood is elevated, which are known to be associated with the progression of CVD (179). These miRNA expression levels were strongly connected with pre-radiotherapy control and post-radiotherapy and adversely correlated with age. Moreover, CVD-related risk variables such as dyslipidemia, therapy being taken, and one side of breast cancer, affected alterations in their levels. These findings may suggest that alterations in these miRNAs may be linked to the progression of radiotherapy-induced CVD in patients with BC. In patients with non-small cell lung cancer (NSCLC) who received irradiation in an investigation, pretreatment c-miRNA signatures were found to be predictive of radiation-induced cardiotoxicity grade 3 or above (G3+) (178). The signatures consisted of 14 c-miRNA species whose serum concentrations changed as G3+ radiation-induced cardiotoxicity progressed.

A recent study screened mouse cardiac tissue for the changes in mRNAs, miRNAs, and lncRNAs levels that predict radiation-induced heart damage after exposing mice to 1, 2, 4, 8, and 12 Gray (Gy) whole-body irradiation for 48 h (177). The study found that the expression alterations in numerous mRNAs and ncRNAs at different doses, such as LncRNA Abhd11os, Pvt1, Trp53cor1, and Dino, rose as the radiation dose increased. Surprisingly, significant upregulation of miRNAs was only found at 1 and 12 Gy, and no miRNAs were detected to be consistently upregulated or downregulated at all doses. This is likely owing to the early time point and rigorous statistical analysis.

### Targeted molecular drugs

In patients with BVZ-treated colorectal cancer, both plasma miR1254 and miRNA579 expression levels were raised, and miRNA1254 was unaffected by different patient characteristics and corresponded most strongly with the clinical diagnosis of BVZ-induced cardiotoxicity (181). This suggests that the specific elevations of miR1254 and miRNA579 may be the important indicators of BVZ-induced cardiotoxicity, allowing BVZ-induced cardiotoxicity to be distinguished from other cardiovascular disorders. Another research of patients with NSCLC receiving BVZ chemotherapy discovered that blood miR-30c expression rose with treatment duration and was positively correlated with cardiotoxicity before and during chemotherapy (180). According to a ROC curve investigation evaluating the area under curve sensitivity and specificity of changes in blood miR-30c levels, miR-30c may be a sensitive biomarker for diagnosing cardiotoxicity in patients with NSCLC treated with BVZ.

## ncRNAs as therapeutic targets in cancer therapy-induced cardiotoxicity

Numerous studies have revealed that ncRNAs play a role in the molecular signal pathways regulating cancer therapy-induced cardiotoxicity. As a result, ncRNAs are being considered potential therapeutic targets for cancer therapy-induced cardiotoxicity, with therapeutic strategies targeting ncRNAs showing promise. Further studies looked at miRNA antagonists and mimic to boost or suppress miRNA expression. For example, as we discussed above, therapeutic silencing of miR-34a expression levels by antimiR-34a and miR-29b overexpression after miR-29b agomir treatment attenuated DOX-induced cardiotoxicity in rats (39, 51). In addition, viral vectors (e.g., adenovirus, AAV, and lentivirus) can be employed to transport ncRNAs to cells and boost their expression levels. For instance, AAV9-based overexpression of miR-200a and lentiviral overexpression of CMDL-1 prevented DOX-induced cardiomyocyte injury, and lentiviral overexpression of circFOXO3 reduced radiation-induced DNA damage and apoptosis (34, 79, 88). Furthermore, siRNA and antisense nucleotides are used to silence ncRNAs, such as knockdown of SOX2-OT and circSKA3, to enhance cardiac function (71, 84).

Cell-based treatments, particularly MSCs, hold considerable promise for the treatment of cardiovascular diseases because of their regeneration capabilities and established biosafety (185). MSCs-based therapies can ameliorate DOX-induced cardiac aging, according to a recent study, and further research has linked MSCs antiaging properties to the regulation of the miR-34a-SIRT1 axis (53). However, stem cell therapy is limited by inconsistent supply, infusion toxicity, poor survival, and immune rejection. Excitingly, exosome-based cell-free therapy is considered to be a promising therapeutic approach, especially MSC-sEVs due to their regenerative and remodeling abilities for cardiac injury (186). Several recent studies have discovered that stem cell-secreted miRNA-rich exosomes can suppress DOX-induced oxidative stress, inflammatory responses, fibrosis, and apoptosis, hence functioning as cardioprotective agents (14, 28, 64). Another study found that MSCs-derived exosomal hypoxia-derived lncRNA MALAT might improve mitochondrial metabolism and promote regeneration in a synergistic manner, ultimately alleviating cardiac aging (80). Notably, the latest study proposes a new local delivery technique based on exosomes and ultrasound, which improves cardiac drug delivery efficiency dramatically (55). Exosomes containing miRNA mimics were administered into experimental animals, and ultrasound-targeted microbubble destruction was done in the heart area. As a result, exosome-mediated miRNA distribution to the heart was greatly enhanced, successfully lowering DOX-induced cardiotoxicity. Therefore, further research is required to generate repeatable and stable MSC-EV products for clinical applications, as the current studies have not looked at clinically meaningful doses.

Several medications have been demonstrated to regulate ncRNA production and signaling pathways to reduce cardiotoxicity caused by cancer therapy, thereby exerting cardioprotective effects. For example, by regulating the miR-30e/Beclin 1 signaling route, ACE2 suppresses DOX-induced cardiac autophagy, whereas dexrazoxane prevents cardiomyocyte death *via* controlling the miR-17-5p/PTEN signaling network (17, 50). In addition, herbal medicines have cardioprotective benefits against cancer-related cardiotoxicity. Chinese medicine Salvia miltiorrhiza's active ingredient, SalA, for instance, is involved in reducing lncRNA PVT1 expression; the Chinese medicine formula Shenmai injection, on the other hand, can control the miR-30a/Beclin 1 pathway and ameliorate heart injury (48, 69). To summarize, ncRNAs have been discovered to have therapeutic promise, but current studies are still in the preclinical stage, and more research is needed in the future.

## Conclusion

One of the most hazardous side effects of cancer treatment is cardiotoxicity, which seriously affects cancer patients' quality of survival. Recently, research on ncRNAs in cancer therapy-induced cardiotoxicity has made substantial progress and is regarded as a promising actor with promising application prospects. This review outlines recent developments in the study of the ncRNAs involved in cancer therapy-induced cardiotoxicity. Apoptosis, mitochondrial damage, oxidative stress, DNA damage, inflammation, autophagy, aging, calcium homeostasis, vascular homeostasis, and fibrosis are all regulated by ncRNAs and play a role in the occurrence and progression of cancer therapy-induced cardiotoxicity. Circulating ncRNAs are being evaluated as potential new biomarkers for detecting cancer therapy-induced cardiotoxicity early, which is crucial for a better patient prognosis. Because circulating ncRNAs are highly stable and specific, tests for them are inexpensive, rapid, and noninvasive, and changes in their expression levels may be beneficial for detecting cardiotoxicity in patients with cancer. Furthermore, ncRNAs have therapeutic potential, with numerous studies confirming that ncRNAs may be used as therapeutic targets to relieve the cardiotoxicity caused by cancer therapy.

To date, researchers have carried out a large number of studies on miRNAs, while understanding of lncRNAs and circRNAs is still in the preliminary exploration stage. There is also a lack of studies on ncRNAs in cardiotoxicity induced by other cancer treatments, such as platinum and paclitaxel used in first-line chemotherapy, and more work focusing on these areas is still needed in the future. In addition, despite the prospective clinical applications of ncRNAs and the great progress gained, many challenges and limits remain. First and foremost, the fundamental problems of ncRNA-based therapeutics are dependable delivery systems and potential off-target consequences, with tissue-specific delivery with long-term on-target effects being a major concern. Second, because cancer therapy-induced cardiotoxicity impacts the expression of numerous ncRNAs implicated in different signaling pathways simultaneously, ncRNAs as therapeutic targets may lack therapeutic specificity, limiting the comprehension of the pharmacological mechanism. Another disadvantage is that most research is conducted on healthy cells or animal models. However, healthy cardiomyocytes cannot represent patients of all ages, and there are species differences in ncRNAs between experimental animals and human clinical environments. Tumors themselves may lead to differential expression of ncRNAs, even among patients from different tumor types. Tumor patient therapy is a dynamic process accompanied by dynamic ncRNA expression. In addition, it is unclear whether ncRNA therapy promotes tumor growth or impacts the effectiveness of cancer treatment. To address these challenges, more research is required in the future. In conclusion, the study of ncRNAs in cancer therapy-induced cardiotoxicity not only contributes to a deeper grasp of the molecular pathways underlying oncological cardiology but also offers a new promising detection method and therapeutic strategy, giving cancer therapy-induced cardiotoxicity patients new hope.

## Author contributions

YX and ZS designed this study. WS wrote the first draft of this manuscript and created figures. JX, LW, YJ, and JC participated in discussions and improved pictures related to the manuscript. XS, FY, and LT critically revised the manuscript. All authors contributed to the article and approved the submitted version.

## Funding

This work was supported by the National Natural Science Foundation of China (Grant Nos. 81725024 and 81430098), the CACMS Innovation Fund (Grant No. CI2021A00919), and the National Key R&D Program of China (Grant Nos. 2018YFC1704901 and 2018YFC1704900).

## Conflict of interest

The authors declare that the research was conducted in the absence of any commercial or financial relationships that could be construed as a potential conflict of interest.

## Publisher's note

All claims expressed in this article are solely those of the authors and do not necessarily represent those of their affiliated organizations, or those of the publisher, the editors and the reviewers. Any product that may be evaluated in this article, or claim that may be made by its manufacturer, is not guaranteed or endorsed by the publisher.
